# Interleukin 16 and 25 (IL-17E) and Clinical Outcomes in Exacerbation of COPD—A Pilot Study

**DOI:** 10.3390/jcm13175188

**Published:** 2024-09-01

**Authors:** Tomasz Karauda, Joanna Miłkowska-Dymanowska, Anna Kumor-Kisielewska, Wojciech J. Piotrowski, Adam J. Białas

**Affiliations:** 1 Department of Pneumology, Medical University of Lodz, 90-419 Lodz, Poland; joanna.milkowska-dymanowska@umed.lodz.pl (J.M.-D.); anna.kumor@umed.lodz.pl (A.K.-K.); wojciech.piotrowski@umed.lodz.pl (W.J.P.); adam.bialas@umed.lodz.pl (A.J.B.); 2 Department of Pulmonary Rehabilitation, Regional Medical Center for Lung Diseases and Rehabilitation, Blessed Rafal Chylinski Memorial Hospital for Lung Diseases, 91-520 Lodz, Poland

**Keywords:** chronic obstructive pulmonary disease, COPD, IL-16, IL-25, survival analysis, immunopathology of COPD

## Abstract

**Background:** Exacerbation of chronic obstructive pulmonary disease (ECOPD) significantly impact health status, hospitalization rates, and disease progression, and are linked to increased mortality. Predictive factors for ECOPD are therefore of considerable interest. The limited understanding of interleukin 16 (IL-16) and IL-25 role in ECOPD provided the rationale for this study. **Methods**: Fifty ex-smokers diagnosed with COPD (22 ECOPD and 28 patients in the stable phase of the disease) underwent prospective analysis to evaluate the role of IL-16 and IL-25 as predictive markers of clinical outcomes in ECOPD. **Results:** We observed a significantly lower IL-16 and higher IL-25 concentrations among ECOPD patients (*p* = 0.002 and *p* = 0.01 respectively). We also detected a significant negative correlation between IL-16 and neutrophil-to-lymphocyte ratio (NLR) (*p* = 0.04) and a significant negative correlation between IL-25 concentration and absolute eosinophil count (*p* = 0.04). In the entire group, we observed a positive correlation between IL-16 and both FEV1 and FVC, both expressed as a percentage of reference value, (*p* = 0.002 and *p* = 0.0004 respectively). However, after stratification to ECOPD and stable COPD group, significance maintained for FVC (*p* = 0.045 for ECOPD and *p* = 0.02 for stable COPD). In survival analysis, we detected significantly lower all-cause mortality for 3rd tertile of IL-16 concentrations, with a hazard ratio of 0.33 (95%CI: 0.11–0.98; *p* = 0.04). **Conclusions**: Lower IL-16 levels among ECOPD patients may indicate a feedback mechanism linked to heightened Th1 response activation. Observed correlations with ventilatory parameters and survival also seems to reflect this mechanism. The higher IL-25 concentrations observed in ECOPD patients, along with the negative correlation with absolute eosinophil count and eosinopenia, suggest multifactorial regulation and independent functions of eosinophils and IL-25. Hypothetically, this paradox may be related to the Th1/Th2 imbalance favoring Th1 response. Obtained results should be reproduced in larger size samples.

## 1. Introduction

Exacerbations of chronic obstructive pulmonary disease (ECOPD) represent significant events in the disease’s natural history, adversely affecting health status, hospitalization rates, and disease progression [1,2]. Additionally, ECOPD necessitating hospitalization are linked to increased mortality [3]. Consequently, research into predictive factors within this cohort is of considerable interest.

Building on existing evidence [4,5], we have previously examined certain aspects of eosinopenia in relation to survival in ECOPD patients [6,7]. However, due to the low specificity of eosinopenia and its rather indirect effects, we extended our research by investigating eosinophil-related cytokines involved in the immunological landscape of COPD. Specifically, we chose to explore the role of interleukin 16 and 25 (IL-25) in this context. Although both interleukins have been previously studied in COPD, their role in the disease’s pathophysiology remains still underexplored.

IL-16 is reported to be a potent chemotactic factor for eosinophils, as well as for CD4+ T lymphocytes [8,9,10]. A study of 152 COPD patients and 80 controls suggested lower concentrations of IL-16 in plasma of males with COPD [11]. Interestingly, a large cohort study showed that low IL-16 plasma and peripheral blood mononuclear cells mRNA expression are associated with emphysema independently from other confounding variables such as age, gender, and BMI [12]. Indeed, some previous evidence also suggested a role for CD4+-mediated autoimmunity in the pathogenesis of emphysema [13]. However, to our knowledge, concentrations of IL-16 in the context of ECOPD has not been examined yet.

IL-25, also known as IL-17E, is a member of the IL-17 cytokine family. It binds to the IL-17RA/IL-17RB receptor complex, activating multiple downstream signaling pathways that may influence cell survival and inflammation [14]. The role of IL-25 in allergic inflammation is well-documented [15,16,17], positioning it as one of key mediators in allergic asthma [18]. However, Th2-mediated immunological responses are observed not only in asthma but also in COPD. Interestingly, in one study, high plasma levels of IL-25 have been associated with positive outcomes in COPD, correlating with a reduced risk of future moderate-to-severe ECOPD [19].

Therefore, the limited understanding of the role of IL-16 and IL-25 in COPD pathophysiology, particularly during ECOPD, provided the rationale for this study.

## 2. Materials and Methods

Ex-smokers diagnosed with COPD (COPD-C ethiotype according to GOLD [1]), referred to the Department of Pneumology at Barlicki Memorial Hospital in Lodz, Poland, as well as to the adjacent outpatient clinic, underwent prospective analysis to evaluate the role of IL-16 and IL-25 as predictive markers of clinical outcomes in COPD exacerbations. In all, the study population consisted of patients diagnosed with COPD (confirmed by documented spirometry with features of non-reversible airway obstruction defined as post-bronchodilator FEV_1_/FVC < 0.7). The study group was divided into patients in the period of acute exacerbation of COPD (category E according to GOLD 2023) and patients in the stable phase of the disease (category B according to GOLD 2023), who had not experienced an exacerbation in the six months prior to enrollment. Patient enrollment occurred between November 2019 and January 2023.

The exclusion criteria were as follows: systemic steroid therapy within 7 days before qualification, active smoking (patients who had not smoked for more than 6 months were considered former smokers), and COPD coexisting with asthma or other chronic lung disease such as idiopathic pulmonary fibrosis, sarcoidosis, primary pulmonary hypertension. Additionally, we excluded patients with confirmed α-1-antitrypsin deficiency, a history of malignant tumors, severe liver failure, any type of diabetes mellitus, severe chronic heart failure (NYHA IV) or exacerbation of chronic heart failure, unstable ischemic heart disease, and acute coronary syndrome within the past year.

At the time of admission, 30 mL of peripheral venous blood was collected from patients into an EDTA tube before systemic steroid administration to avoid pharmacology-induced anti-inflammatory effects. Blood was drawn during routine tests by nursing staff for patients experiencing an exacerbation of COPD, and during outpatient visits as an additional procedure by the principal investigator or a co-investigator. As mentioned previously, only patients who had not undergone any systemic steroid therapy within 7 days before qualification were included. Routine blood tests (complete blood count, c-reactive protein) were examined with an automated analyzer.

To quantify the concentration of IL-25 in the samples, we employed enzyme-linked immunosorbent assays (ELISAs). The ELISA kits were purchased from Cloud-Clone Corp (Houston, TX, USA). The assays were performed following the manufacturer’s instructions. The concentrations of IL-25 and IL-16 in the samples were determined by comparing the absorbance values to the standard curves generated for each cytokine.

Spirometry was performed using a Lungtest 1000 system (MES, Cracow, Poland) in accordance with ATS/ERS standards [20].

Statistical analysis was conducted using R software version 4.2.3 for MacOS. Continuous data were presented as means with standard deviations (SDs) or medians with interquartile ranges [IQRs], depending on data distribution. Between-group comparisons utilized the unpaired Student’s *t*-test, Welch *t*-test, or Wilcoxon rank-sum test, based on data normality and variance homogeneity. Categorical data were analyzed using Pearson’s Chi-squared test or Fisher’s Exact Test, depending on the test assumptions. Missing data were not imputed in the analysis. Corrections for multiple comparisons were not applied due to the predefined hypotheses and comparisons specified before data collection. The survival analysis for IL-25, as there is no wide-accepted, established cut-off values for IL-16 or IL-25, was conducted after dividing the concentrations into tertiles. The survival curves were estimated using the Kaplan–Meier method, and the logrank test was applied to assess the statistical significance of differences between subgroups. The post-hoc power for the comparison of IL-16 and IL-25 between ECOPD and the stable phase was estimated using G*Power software 3.1.9.6 for MacOS [21].

The study protocol was approved by the Bioethical Committee of the Medical University of Lodz (RNN/359/19/KE). Informed consent was obtained from each patient.

## 3. Results

### 3.1. Baseline Data Analysis

A total of 50 patients were enrolled in the study: 22 (44%) patients diagnosed with ECOPD, and 28 (56%) patients in a stable period of the disease. Baseline study data are presented in Table 1.

### 3.2. ECOPD Group

We enrolled 22 patients with ECOPD who had not received systemic steroids before blood sample collection. We observed a significantly lower IL-16 concentration and higher IL-25 concentration among ECOPD patients (*p* = 0.002; post-hoc 1-*β* = 0.9 and *p* = 0.01; post-hoc 1-*β* = 0.72 respectively, see Table 1). The groups differed significantly in terms of arterial hypertension (HA) and heart failure (HF). None of the recruited patients showed a significant probability of pulmonary hypertension. There were no significant differences in IL-16 concentrations between patients with and without HA (*p* = 0.96) or HF (*p* = 0.66), nor in IL-25 concentrations (*p* = 0.43 for HA and *p* = 0.87 for HF). Baseline characteristics of the ECOPD patients are presented in Table 2.

We did not observe any significant correlation between IL-16 and WBC (*p* = 0.52), CRP (*p* = 0.17), or length of hospitalization (*p* = 0.14). However, a significant negative correlation was identified with NLR (*p* = 0.04).

**Figure 1:**
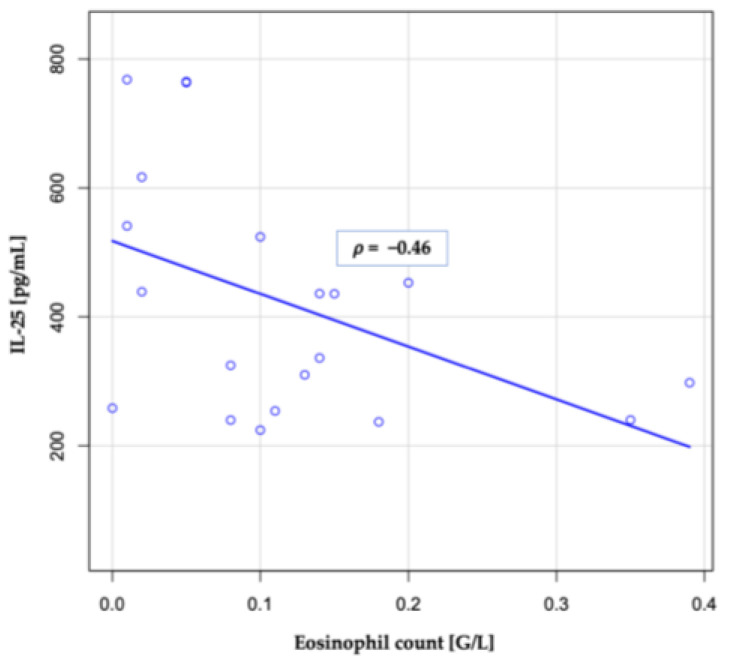
Correlation between eosinophil count and IL-25 concentrations in ECOPD group.

No significant correlation was found between IL-25 and WBC (*p* = 0.87), NLR (*p* = 0.23), CRP (*p* = 0.06), or length of hospitalization (*p* = 0.27). However, a significant correlation was observed between IL-25 concentration and absolute eosinophil count (*p* = 0.04; Figure 1). Additionally, patients with eosinopenia (defined as an eosinophil count ≤ 0.5 G/L [22]) exhibited significantly higher concentrations of IL-25: 593.11 (195.1) vs. 331.69 (99.48) (*p* = 0.0009).

In our cohort, no significant relationships were observed between the analyzed cytokines and lymphocyte or neutrophil count.

### 3.3. IL-16, IL-25 and Spirometry Results

**Figure 2:**
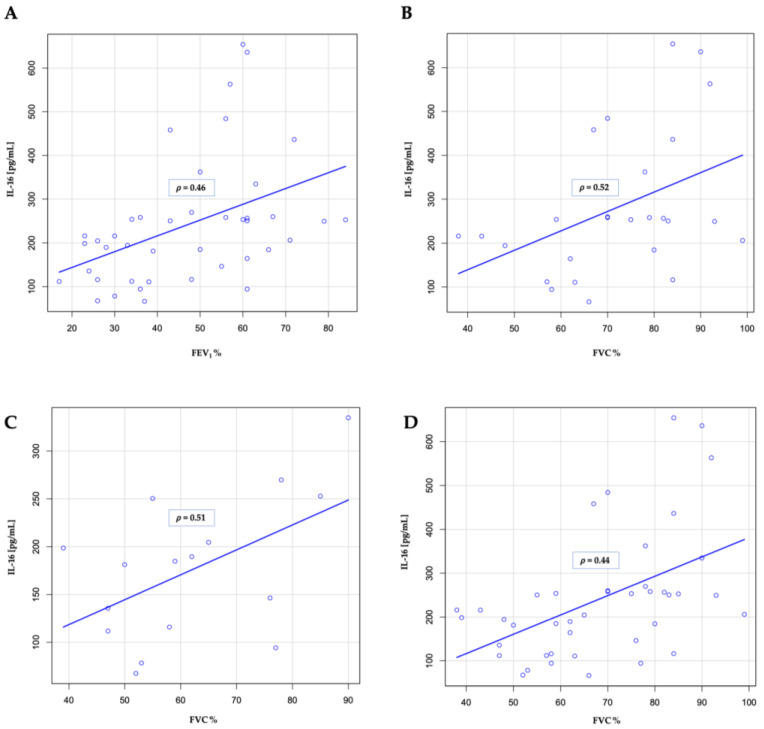
Correlations between FEV1 and FVC (both expressed as percentages of the reference value) and IL-16 concentrations (**A**,**B**) in the entire group. Correlations between FVC and IL-16 concentrations after stratification into ECOPD (**C**) and the stable phase of the disease (**D**). The blue lines represent least-squares lines. Spearman’s rank correlation coefficients (*ρ*) are shown in the boxes near the blue lines.

In our study, in the entire group, we observed a positive correlation between IL-16 and both FEV_1_ and FVC, both expressed as a percentage of reference value, (*p* = 0.002 and *p* = 0.0004 respectively; Figure 2A,B). However, after stratification to ECOPD and stable COPD group, significance maintained for FVC (*p* = 0.045 for ECOPD and *p* = 0.02 for stable COPD; Figure 2C,D).

We did not observe a significant correlation between IL-25 and either FEV1 (*p* = 0.37) or FVC (*p* = 0.33).

### 3.4. All-Cause Survival

**Figure 3:**
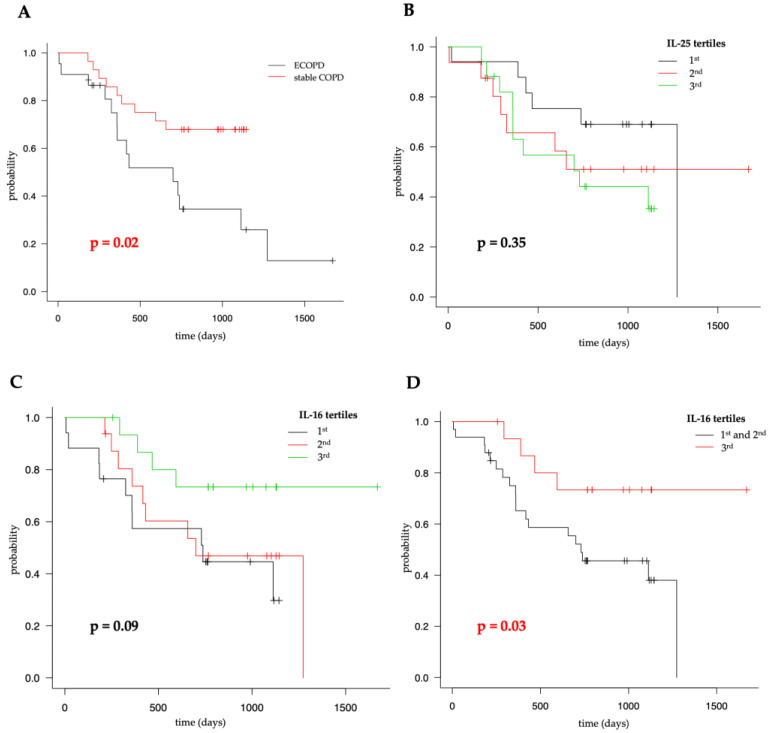
Kaplan–Meier curves for the difference in survival between ECOPD group and patients in stable phase of the disease (**A**), IL-25 tertiles (**B**), IL-16 tertiles (**C**), and for extracted 3rd tertile (**D**).

We did not observe any cases of in-hospital death. However, as anticipated, patients with ECOPD showed significantly higher all-cause mortality (*p* = 0.02; see Figure 3A). Additionally, in the analyzed group, eosinopenia was associated with worse survival (*p* = 0.02) with a hazard ratio of 3.92 (95%CI: 1.09–14.14; *p* = 0.04).

We analyzed the entire group of patients by dividing IL-16 and IL-25 concentrations into tertiles (IL-16: T1 < 147.92 pg/mL, T2 147.92–252.78 pg/mL, T3 > 252.78 pg/mL; IL-25: T1 < 265.82 pg/mL, T2 265.82–438.04 pg/mL, T3 > 438.04 pg/mL). We did not observe any differences in survival between IL-25 subgroups (see Figure 3B). However, we detected a trend among IL-16 subgroups, particularly in the third tertile, showing a visible separation (see Figure 3C). Therefore, we combined the first and second tertiles versus the third tertile, achieving statistical significance for better survival in this subgroup (see Figure 3D), with a hazard ratio of 0.33 (95%CI: 0.11–0.98; *p* = 0.04).

The group was too small to conduct reliable analysis in subgroups.

## 4. Discussion

To our knowledge, this is a first study to investigate IL-16 and IL-25 concentrations during ECOPD. Our results showed significant differences among analyzed groups in this context. Namely, lower IL-16 and higher IL-25 concentrations.

Lower IL-16 concentration among ECOPD patients might signify a feedback mechanism triggered by heightened Th1 response activation. This observation aligns with substantial evidence highlighting the prevalence of Th1 dominance in COPD patients [23,24], as well as an imbalance of Th1/Th2 cells favoring the Th1 cell-mediated immune response in ECOPD [25]. Th1 response dominance, as being linked to severity of lung destruction in COPD [13,26] seems also to justify the detected correlation between IL-16 and ventilatory parameters. However, given that IL-16 facilitates the secretion of multiple proinflammatory cytokines and exerts chemoattractant activity toward eosinophils and CD4+ T-cells [8,9,10,27,28]—both of which contribute to the production of various pro-inflammatory cytokines—this observation warrants further investigation within a broader context before definitive conclusions can be made. Also, the role of IL-16 as a potential predictor of survival requires more comprehensive and in-depth exploration.

Additionally, we also found a notable inverse relationship between IL-16 and NLR in ECOPD group. NLR has been extensively studied across diverse clinical settings and diseases, including COPD. It demonstrates significant correlations and, in certain studies, even exhibits superiority to traditional inflammatory markers like WBC and more complex and costly biomarkers such as CRP [29]. However, such a negative relationship also seems to reflect indirect mechanisms and deserves further, more detailed research in larger sizes.

Higher concentrations of IL-25 among ECOPD patients can be justified by the release of this cytokine, among other alarmins, in response to epithelial activation by irritants such as pathogens or air pollutants [30]. However, the observation of its negative correlation with the absolute eosinophil count and its higher concentrations among patients presenting eosinopenia is intriguing. One of the possible explanations of this is the apparent paradox that eosinopenia in ECOPD is a multifactorial phenomenon and may suggest differential and independent functions for eosinophils and IL-25 in ECOPD. Hypothetically, this paradox may be also associated with the aforementioned imbalance of Th1/Th2 cells favoring the Th1 cell-mediated response. Stringent exclusion criteria of our study included systemic steroid therapy within 7 days before qualification, avoiding the depletory effect of these drugs on peripheral eosinophil count. However, we cannot exclude such an effect induced by endogenous steroids such as cortisol. There are also other contributing mechanisms including migration of eosinophils to the site of inflammation, rapid peripheral sequestration, suppression of egress of mature eosinophils from the bone marrow, and suppression of eosinophils production [31,32]. Therefore, these issues also require further investigation.

The primary limitation of our study is the relatively small sample size, which characterizes it as exploratory and hypothesis-generating. This limitation stems largely from stringent exclusion criteria implemented to minimize the impact of external confounders on the inflammatory processes in ECOPD, ensuring that the observed effects were not influenced by comorbidities. Specifically, it was challenging to enroll patients who had not received systemic steroids prior to blood sample collection for analysis. Moreover, the SARS-CoV-2 pandemic, which began during the recruitment period, introduced significant administrative challenges that further impacted study enrollment. As a result, we opted to conclude the study and publish it as a pilot study, having achieved satisfactory post-hoc power for the main endpoint, specifically the comparison of IL-16 and IL-25 between ECOPD and the stable phase. Given the statistical significance and the novel insights provided, we believe that, even as a pilot study, our preliminary findings are valuable to the field and warrant further investigation. Additionally, the study was conducted at a single institution, which may limit the generalizability of the findings.

## 5. Conclusions

This study represents the first investigation into IL-16 and IL-25 concentrations during ECOPD. The findings suggest distinct roles and regulatory mechanisms for these cytokines in the immunopathology of ECOPD, which warrant further research in this field. Lower IL-16 levels among ECOPD patients may indicate a feedback mechanism linked to heightened Th1 response activation. The observed correlations with ventilatory parameters and survival also support this mechanism. On the other hand, higher IL-25 concentrations in ECOPD patients, along with the negative correlation with absolute eosinophil count and eosinopenia, suggest multifactorial regulation and independent functions of eosinophils and IL-25. Hypothetically, this paradox may also be related to a Th1/Th2 imbalance favoring the Th1 response. Obtained results underscore the need to replicate these results in larger sample sizes.

## Figures and Tables

**Table 1:** Baseline data analysis. Abbreviations: BMI—body mass index, FEV_1_—forced expiratory volume in the first second, FVC—forced vital capacity, IL—interleukin, IQR—interquartile range, SD—standard deviation.

Parameter	ECOPD N = 22	Stable COPD N = 28	Total N = 50	*p*-Value
Age, years, mean (SD)	74.14 (7.03)	72.54 (7.66)	73.24 (7.36)	0.45
Male sex, n (%)	13 (59.09)	10 (35.71)	23 (46)	0.1
BMI, kg/m^2^, mean (SD)	27.08 [23.32–28.62]	24.79 [21.74–28.82]	26.35 [21.74–28.82]	0.64
***Major comorbidities***				
Arterial hypertension	18 (81.82)	15 (53.57)	33 (66)	0.04
Ischemic heart disease, n (%)	5 (22.73)	6 (21.43)	11 (22)	1.0
Atrial fibrillation, n (%)	5 (22.73)	3 (10.71)	8 (16)	0.28
Heart failure, n (%)	9 (40.91)	4 (14.29)	13 (26)	0.03
Overweight and obesity, n (%)	11 (50)	12 (42.86)	23 (46)	0.15
Chronic kidney disease, n (%)	1 (4.55)	1 (3.57)	2 (4)	1.0
Depression	2 (9.09)	3 (10.71)	5 (10)	1.0
***Spirometry***				
FEV_1_, %, mean (SD)	40.41 (18.07)	51.15 (15.19)	46.91 (17.03)	0.04
FVC, %, mean (SD)	63.18 (15.24)	72.08 (15.65)	68.56 (15.92)	0.07
***Study parameters***				
IL-16, pg/mL, median [IQR]	146.34 [94.38–198.54]	251.9 [179.36–359.22]	198.54 [116.33–258.11]	0.002
IL-25, pg/mL, mean (SD)	451.56 (202.09)	306.92 (190.43)	370.56 (206.75)	0.01

**Table 2:** Baseline data analysis of patients with exacerbation of COPD. Abbreviations: IL—interleukin, IQR—interquartile range, NLR—neutrophil-to-lymphocyte ratio, SD—standard deviation, WBC—white blood count.

Parameter	Result
Length of hospitalization, days, median [IQR]	4 [1–5.75]
WBC, G/L, median [IQR]	8.3 [7.5–9.7]
Neutrophils, G/L, median [IQR]	5.5 [4.9–7.23]
NLR, median [IQR]	4.57 [3.16–9.66]
Lymphocytes, G/L, median [IQR]	1.35 [1–1.58]
Eosinophils, G/L, median [IQR]	0.1 [0.04–0.14]
Eosinopenia, n (%)	5 (22.73)
Hemoglobin, g/dL, mean (SD)	13.95 (1.52)
Platelets, G/L, mean (SD)	258.32 (74.82)
C-reactive protein, mg/L, median [IQR]	4.85 [3–40.68]
IL-16, pg/mL, median [IQR]	146.34 [94.38–198.54]
IL-25, pg/mL, mean (SD)	451.56 (202.09)

## Data Availability

The data presented in this study are available from the corresponding author upon reasonable request.
